# Tick-borne encephalitis virus NS4A ubiquitination antagonizes type I interferon-stimulated STAT1/2 signalling pathway

**DOI:** 10.1080/22221751.2020.1745094

**Published:** 2020-03-27

**Authors:** Qi Yang, Jia You, Yuan Zhou, Yun Wang, Rongjuan Pei, Xinwen Chen, Min Yang, Jizheng Chen

**Affiliations:** a Department of Gastroenterology, Guangzhou Women and Children’s Medical Center, Guangzhou, People’s Republic of China; b State Key Laboratory of Virology, Wuhan Institute of Virology, Center for Biosafety Mega-Science, Chinese Academy of Sciences, Wuhan, People's Republic of China; c College of Pharmacy and State Key Laboratory of Medicinal Chemical Biology, Nankai University, Tianjin, People’s Republic of China; d Guangzhou Institutes of Biomedicine and Health, Chinese Academy of Sciences, Guangzhou, People’s Republic of China

**Keywords:** Tick-borne encephalitis virus (TBEV), NS4A, ubiquitination, STAT1, STAT2

## Abstract

Tick-borne encephalitis virus (TBEV) accounts for approximately 10,000 annual cases of severe encephalitis in Europe and Asia and causes encephalitis in humans. In this study, we demonstrate TBEV appears to activate the interferon (IFN)-β dependent on RIG-I/MDA5. Both the IFN-β accumulation and the IFN stimulated genes (ISGs) transcription greatly delay. Further studies reveal that TBEV NS4A could block the phosphorylation and dimerization of STAT1/STAT2 to affect type I and II IFN-mediated STAT signalling. Additional data indicate that the residue at K132 of TBEV NS4A could be modified by ubiquitination and this modification is necessary for the interaction of NS4A with STAT1. Dynamic ubiquitination of the NS4 protein during TBEV infection might account for delayed activation of the ISGs. These results define the TBEV NS4A as an antagonist of the IFN response, by demonstrating a correlation between the association and STAT interference. Our findings provide a foundation for further understanding how TBEV evade innate immunity and a potential viral target for intervention.

## Introduction

Tick-borne encephalitis virus (TBEV) is a tick-borne flavivirus that causes encephalitis, meningitis and haemorrhagic fevers with mortality rates as high as 20%–30% [1,2]. TBEV is considered one of the most important arboviruses in central and eastern European countries, Russia and northeast China [3,4]. There has been an approximately 300% increase in the incidences of TBEV in Europe [5]. This increase might be due to a growth in the population and the spread of ticks [6,7]. TBEV is a member of the genus *Flavivirus* whose positive-sense RNA genome is transcribed as one large open reading frame and is processed into three structural proteins (envelope [E], membrane [M], and capsid [C] proteins) and seven non-structural proteins (NS1, NS2A, NS2B, NS3, NS4A, NS4B, and NS5) [8]. The mechanism of TBEV infection and pathogenesis remain largely unsolved. For instance, it is not well understood how TBEV regulates the innate immune system, particularly the antiviral type I interferon (IFN) pathway.

The type I IFN pathway promotes a delay in viral replication and the activation of effector cells. Pattern recognition receptors (PRRs) such as RIG-I-like receptors (RIG-I) and Toll-like receptors (TLR), bind viral pathogen-associated molecular patterns (PAMPs), including double-stranded RNA (dsRNA) and 5’-phosphate-containing single-stranded RNA [9]. These PRRs, when activated, convey their signal through the transcription factors IFN regulatory factor 3 (IRF3), NF-κB, and AP1, which act in concert to induce the expression of type I IFN [10]. Secreted type I IFN binds to IFN receptors in an autocrine or paracrine manner stimulating the type I IFN signalling pathway [11]. The signal transducer and activator of transcription (STAT) family comprise transcription factors that are important in innate immune responses. The STAT1 and STAT2 proteins exist as latent monomers in the cytoplasm and become activated by phosphorylation upon IFN-α/β stimulation of the IFNAR-1/2 receptor through tyrosine phosphorylation via the Janus kinases JAK1 and TYK2 [12]. Tyrosine-phosphorylated STAT1 and STAT2 heterodimerize and join with IFN regulatory factor 9 (IRF9) to form the transcription factor complex ISGF3 [13]. ISGF3 translocates to the nucleus and is recruited to specific genetic elements, termed IFN-sensitive response elements (ISREs), located within upstream promoter regions of IFN stimulated genes (ISGs) [14]. ISGF3 activation results in increased levels of expression of over 100 different proteins that function to create an antiviral state within the cell, thus inhibiting viral replication.

The type I IFN pathway plays a key role in the clearance of viral infection. Consequently, viral gene products interfere with induction, signalling and the effects of type I IFNs. Flaviviruses use different proteins to disturb signalling components of the pathway [15–18]. TBEV induces type I IFN, and a few ISGs have been identified to inhibit TBEV replication such as 2’-5’-oligoadenylate synthetase (2’-5’-OAS, OAS), TRIM79α and Viperin [19–21]. The induction of type I IFN is slowed down after infection [22,23]. It has been proposed that TBEV infection rearranges the internal cell membrane structure, which may serve to protect the replication of intermediate dsRNA from host cellular surveillance [23,24]. Even though all TBEV proteins, as well as TBEV infection itself, has no apparent influence on IFN-β induction, the NS5 protein is identified previously as an inhibitor of IFN-dependent signalling [18,22].

In this report, we demonstrated that TBEV NS4A could bind STAT1/STAT2 and inhibit type I IFN-dependent signalling. The NS4A targeted STAT1/STAT2 to inhibit their phosphorylation and dimerization. The NS4A K27-linked ubiquitination at Lys132 is essential to antagonize the signalling. The ability of NS4A to block type I IFN signalling illustrates the major role that this pathway plays in controlling TBEV infections.

## Materials and methods

### Reagents and plasmids

Recombinant human IFNα/β were purchased from R&D Systems. Rabbit anti-Flag, HA, STAT1, phospho-STAT1 (Tyr701), STAT2, phospho-STAT2 (Tyr690), IRF-9, Ub and phospho-Jak family antibody sampler kit were all purchased from Cell Signalling Technology. Mouse anti-Flag and HA were from Sigma-Aldrich. Rabbit anti-IFNAR1 and IFNAR2 were from Abcom. Mouse anti-β-actin antibody was purchased from Proteintech.

JAK1, TYK2, STAT1, STAT2 and IRF9 were amplified from HEK293 T cDNA, and subsequently cloned into mammalian expression vectors as indicated. The deletion and site-directed mutants of STAT1/2 and NS4A were constructed by a PCR-based approach and then cloned into pcDNA3.1(-) or pFlag-CMV2 vectors with HA- or Flag-tag at the N-terminus, respectively. The TBEV NS4A plasmid was kindly provided by Prof. Zongqiang Cui. The *IFNBI* and *ISRE* promoter luciferase reporter plasmids were purchased from Clontech. All primers used in this study are described in Supporting Information.

### Cell culture and plasmid transfection

HEK293 T, THP-1 and T98G cells were obtained from American Type Culture Collection and cultured in the different medium supplemented with 10% fetal bovine serum (FBS) (Invitrogen) at 37°C in a 5% CO_2_ incubator. Plasmids were transfected into cells using Lipofectamine 2000 (Invitrogen) or Lipofectamine 3000 (Invitrogen) following the manufacturer instructions.

### Virus infection

Sendai virus (SeV) and vesicular stomatitis virus VSV-GFP (provided by Prof. Hanzhong Wang), HSV-1 BAC with GFP (provided by Prof. Chunfu Zheng). All virus was amplified and titrated by standard plaque or focus-forming assays. Cells were infected with SeV (1 M.O.I.), VSV (1 M.O.I) or HSV-1 (10 M.O.I.) for the indicated hours.

### Luciferase assay

The dual-Luciferase reporter assay system (Promega) was used for luciferase assays. Briefly, cells were seeded in 48-well plates (5 × 10^4^ cells per well) and transfected luciferase reporter and pRL-TK plasmids combined with a total of 200 ng of target plasmid or empty control plasmid for 24 h. Subsequently, cells were stimulated with IFNα (50 ng/mL), IFNβ (50 ng/mL), IFNγ (100 ng/ml) or left untreated for the indicated time periods and luciferase activity was measured.

### Lentiviral transduction and establishment of stable cell lines

The lentiviruses System (Bought from addgene, http://www.addgene.org) was used for NS4A overexpression assays. In brief, HEK293 T cells were co-transfected with the pWPI-NS4A plasmid and packaging plasmids psPAX2 and pMD2.G in 4:3:1 ratio. 60 h after transfection, the supernatant was collected and applied to infect target cells in the presence of polybrene (8 μg/ml). THP-1 cells served for the generation of cell lines with stably overexpressing TBEV NS4A protein.

### Real-time RT-PCR

Total cellular RNA was isolated with TRIzol (Invitrogen) reagent according to the manufacturer’s protocols. The quantification of *IFNBI*, *ISG15/54/56* transcripts was analysed by one-step real-time RT-PCR with the QuantiFast SYBR Green RT-PCR kit (Qiagen). The data were normalized to levels of β-actin mRNA in each individual sample. 2^−ΔΔCt^ method was used to calculate relative expression changes. The sequences of the primers for quantitative RT-PCR are in Supplementary Table 2.

### ELISA assay

Secreted IFN-β in cell culture supernatants from SeV stimulus 12 h were analysed using human IFN-β (Cusabio Biotechnology) ELISA kit according to the manufacturer’s instructions.

### Coimmunoprecipitations, Western blotting, and ubiquitination assays

Whole-cell lysates were prepared 36 h post-transient transfection in a immunoprecipitation (IP) lysis buffer containing 50 mM Tris, pH 7.5, 1 mM EGTA, 1 mM EDTA, 1% Triton X-100, 150 mM NaCl, 100 µM phenylmethylsulfonyl fluoride (PMSF) and Complete ^TM^ protease inhibitors (Roche Applied Science) for 30 min in 4°C. Cell lysates were centrifuged at 12,000 × g for 10 min at 4°C and quantified using the Bradford method. The immunoprecipitates were subjected to standard Western blotting analysis. For ubiquitination assays, the cells were added 1 µg/ml MG132 2 h before preparing samples and the immunoprecipitates were analysed by Western blotting with antibodies specific for ubiquitin.

### In vitro kinase assay

The tested proteins TYK2, HA-STAT1, HA-STAT2 or Myc-NS4A in Figure 6(A) were in vitro translated with a TNT Quick-coupled Transcription/Translation Systems kit (Promega) following instructions of the manufacturer. Kinase reactions were performed by incubation of 1.0 μg purified HA-STAT1 or HA-STAT2 as the substrate with 1× kinase buffer, 1 mM ATP, immunoprecipitated Myc-NS4A and JAK1 or TYK2 at 30°C for 60 min in 50 μl reaction mixture. Reaction was stopped by addition of 2×SDS loading buffer and samples were separated by SDS-PAGE, and analysed by immunoblotting with anti-phospho-STAT1 or anti-phospho-STAT2.

### Blocking assays with NS4A peptides

The TBEV NS4A residues aa111–130 or residues aa120–134 were synthesized (GL Biochem). The plasmids Flag-STAT1 or HA-STAT2 were transfected into HEK293T cells. Cells were seeded at 2 × 10^5^ cells per ml one day before transfection. Two days after transfection, the whole-cell lysates (1000 μg) were incubated for 4 h at 4°C with anti-HA-agarose or anti-Flag-agarose (Protein G agarose, Millpore). The purified protein was dialysed into 20 mM HEPES, 150 mM NaCl (pH 7.5). After purification, HA-STAT2 and BSA, peptide 111–130 or peptide120–134 were incubated at a 1:10 ratio 2 h at 4°C and then added the purified Flag-STAT1 overnight at 4°C. Reaction was stopped by addition of 2×SDS loading buffer and samples were separated by SDS-PAGE, and analysed by immunoblotting with anti-Flag antibody.

### Statistics

Statistical significance was assigned when *P* values were <0.05 using Prism Version 5 (GraphPad). The results are quantifications from multiple independent experiments and are described in each corresponding figure legend. Quantitative data displayed as histograms are presented as means ± SD (represented as error bars). Data were analysed using a Student’s unpaired *t*-test or multiple *t*-test.

## Results

### TBEV induces IFN-β activation

**Figure 1.**
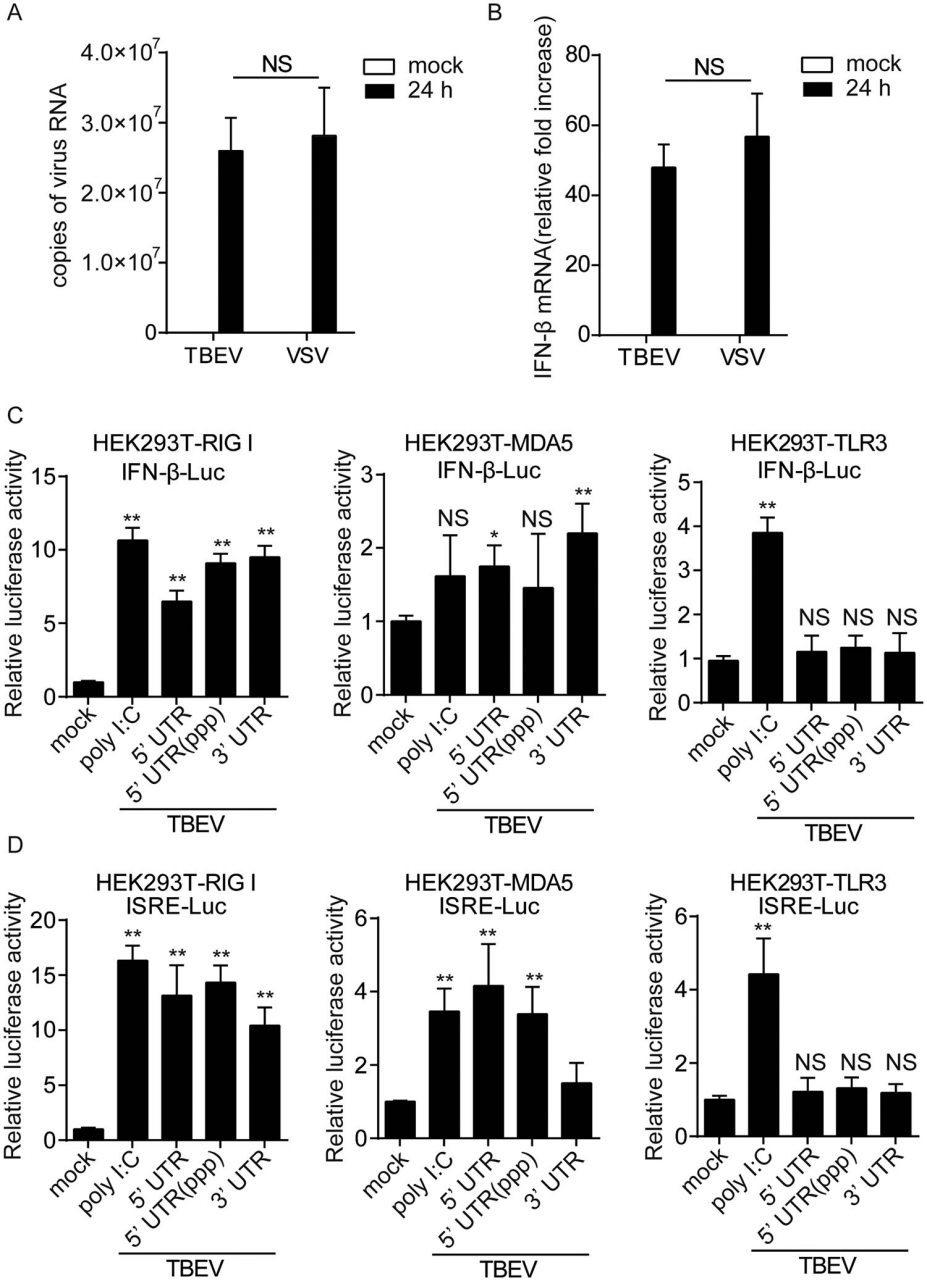
The TBEV induces IFN-β activation. T98G cells were infected with TBEV at an MOI of 1. A control infection was performed with VSV at MOI = 0.1. The cellular RNA was extracted either at 24 h.p.i.. The Viral RNA (A) and IFN-β mRNA levels (B) were quantified by real-time RT-PCR analysis as indicated. β-Actin mRNA quantification from the same samples was used for normalization. The stable cell lines (HEK293T-RIG-I, HEK293T-MDA5 or HEK293T-TLR3, 1×10^5^ cells) were transfected with firefly luciferase reporter plasmid for *Ifnb1* (IFN-β–Luc; C) or ISRE (ISRE-Luc; D) and pRL-TK (Renilla luciferase as an internal control for transfection efficiency, 5 ng), together with 10 µg of UTR RNA from the 5’ end of the TBEV, 3’ end of the TBEV or triphosphorylated of 5’ UTR RNA (5’ UTR (ppp)) of TBEV, the poly I:C as the positive control. It should be pointed out that these three inducers were added to supernatant of HEK293T-TLR3. The reporter activity was detected by dual-luciferase reporter system, luciferase reporter activity is normalized to that of renilla luciferase. Student’s *t*-test was used for estimation of statistical significance. NS, there was no significant difference; *, *P* < 0.05 and **, *P* < 0.01. Data are from three independent experiments. Mean values and standard deviations from three independent experiments per group.

To investigate the interferon response triggered by TBEV RNA, human glioma cells (T98G) were infected with the TBEV (WH-2012) [25]. The vesicular stomatitis virus (VSV) was used as a positive control. As shown in Figure 1(A,B), real-time PCR analyses showed that TBEV infection induced the transcription of IFN-β. Both TBEV and VSV induced similar levels of mRNA at 24 h.p.i. (hours post-infection). Compared with VSV infection, IFN-β induced by TBEV showed an effective activation at the IFN-β mRNA levels, which is consistent with previous reports [22,23].

To explore which molecules within the type I IFN pathway induce IFN-β transcription, we constructed stable expression HEK293 T cell lines of RIG-I-like receptors (RIG-I and MDA5) and Toll-Like receptor 3 (TLR3). The cells were then challenged with the UTR RNA from the 5’ end of the TBEV, 3’ end of the TBEV or triphosphorylated of 5’ UTR RNA (5’ UTR (ppp)) of TBEV. Poly I:C was used as a positive control. As shown in Figure 1(C,D), in IFN-β- and IRES-derived reporter gene assays, the RIG-I and MDA5 but not TLR3 could recognize TBEV RNA to induce ISRE activation and IFN-β upregulation. These results indicate that TBEV induces IFN-β mRNA through RIG-I or MDA5, but not through TLR3.

### TBEV delays IFN-β-induced antiviral effects

**Figure 2.**
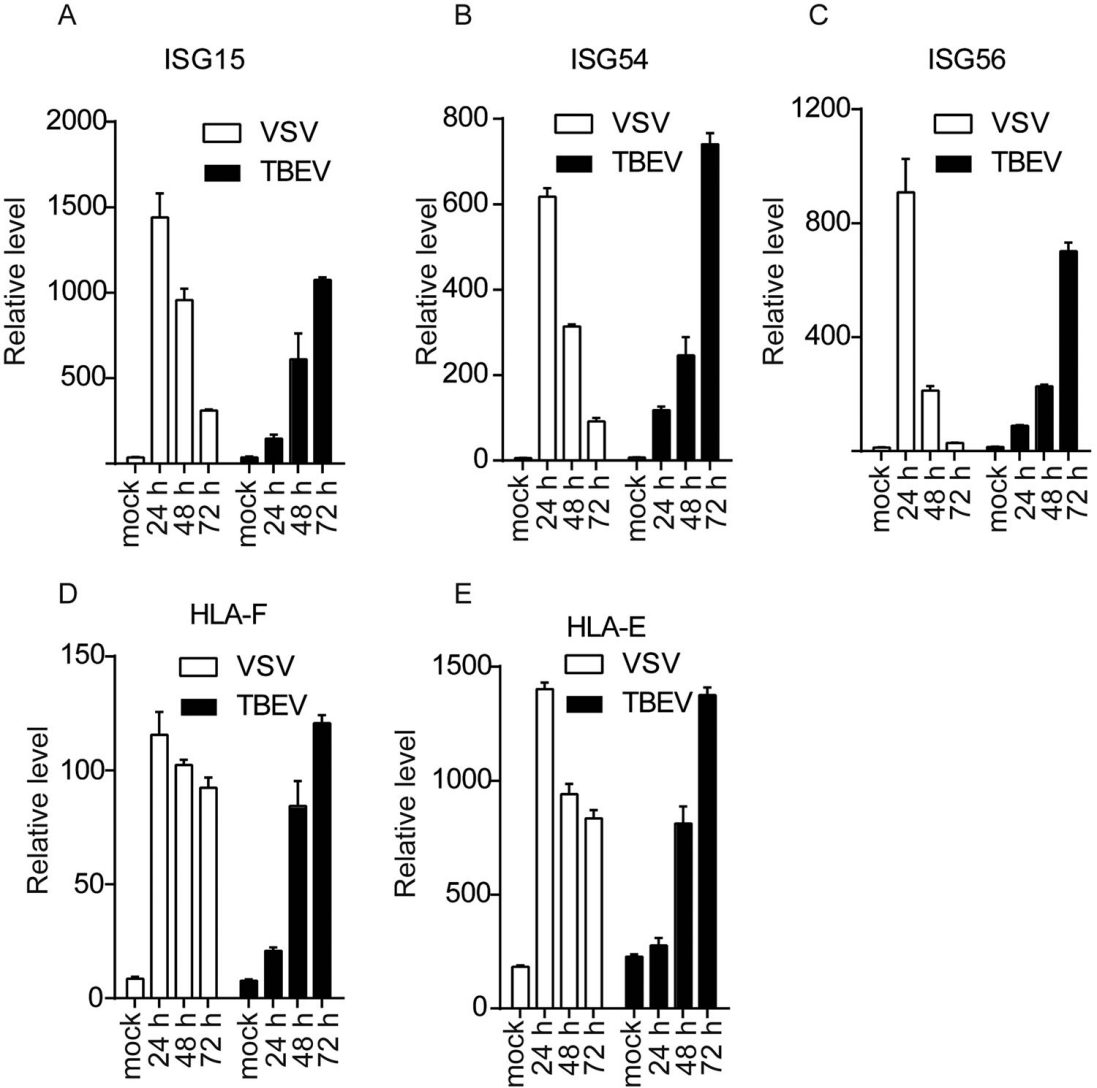
Induction of ISGs in TBEV or VSV infection. (A-E) T98G cells were infected with TBEV at an MOI of 1, and total cell RNA was extracted at indicated time point. Levels of ISG15, ISG54, ISG56, HLA-F and HLA-E mRNAs were measured by real-time RT-PCR analysis, normalized to the cellular β-actin mRNA, and set in relation to mRNA levels of mock-infected cells. A control infection was performed with VSV at an MOI of 0.1. Mean values and standard deviations from three independent experiments are shown.

We had shown an effective upregulation of IFN-β after TBEV infection. We further performed transcriptome sequencing to analyse the ISG-expression response to TBEV infection. T98G cells were infected with TBEV at an MOI of 1 and total RNA was extracted at 48 and 72 h.p.i.. Cellular transcript levels were normalized to mock-infected cells. Supplementary Fig. 1 showed that only a few ISGs were upregulated at 48 h.p.i., while most ISGs were stimulated by several orders of magnitude till 72 h.p.i.. We confirmed the expression of selected classic ISGs in the context of TBEV or VSV infection by real-time PCR (Figure 2(A–F)). The activation of ISG15, ISG54, ISG56 and HLAs could be induced effectively by VSV infection, but the enhanced transcription of the ISGs was further delayed by TBEV infection. The mRNA of ISG15, ISG54, ISG56 or HLAs in TBEV-infected cells reached the highest level at 72 h p.i. Thus, TBEV might interfere with the IFN-β-dependent signalling pathway, leading to delayed ISG expression.

### NS4 inhibits the signalling pathway induced by type I IFN

**Figure 3.**
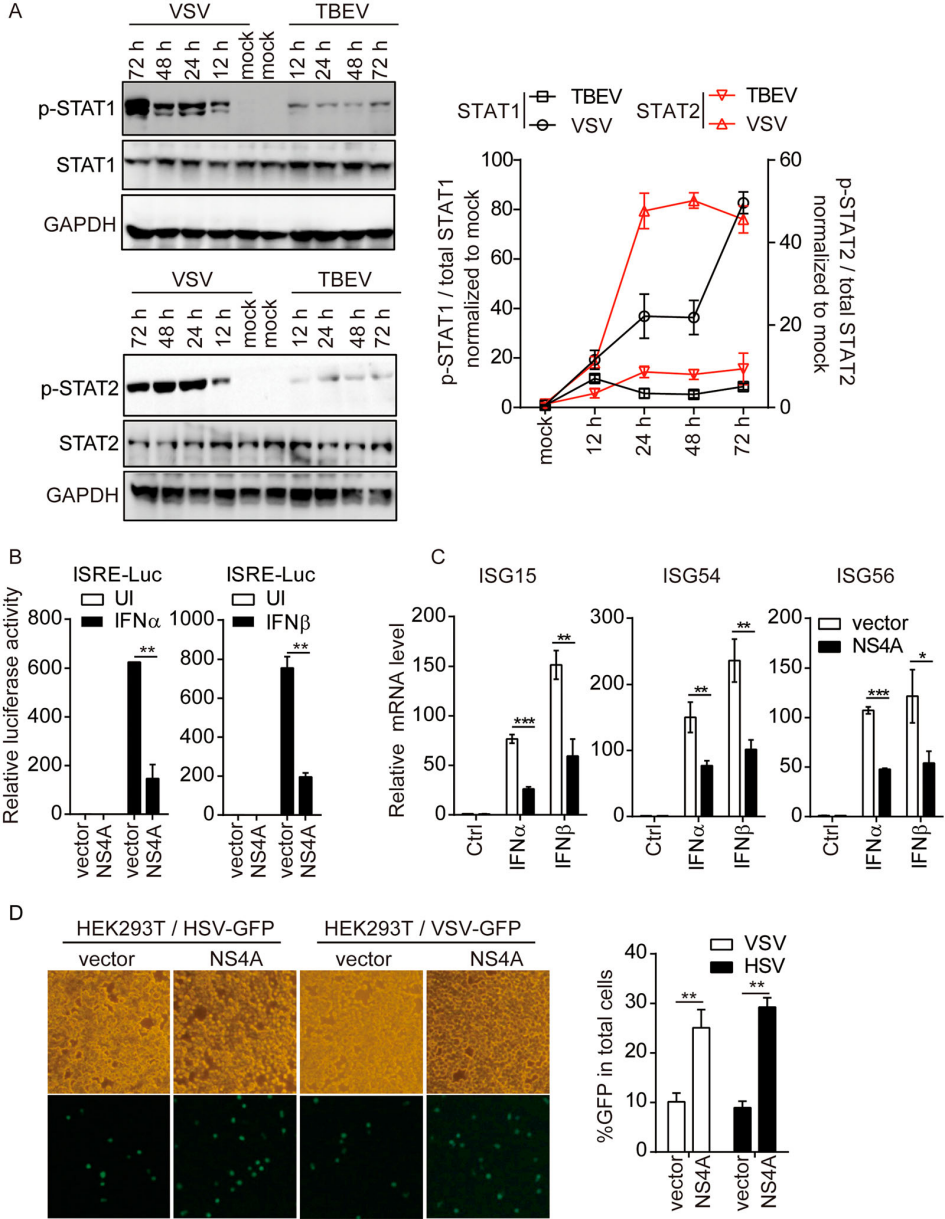
NS4 inhibits the signalling pathway induced by type I IFN. (A) Tyrosine phosphorylation of STATs in TBEV or VSV-infected T98G cells. T98G cells at 1 × 10^6^ infected by TBEV (MOI = 1) or VSV (MOI = 0.1) at various time points were collected and lysed, and the whole lysates were prepared. Western blotting was performed to determine the levels of total and tyrosine-phosphorylated (p-) STAT1 (left, top) and STAT2 (left, bottom) during TBEV or VSV infection. GAPDH was used as a loading control. Histogram (right) representing a densitometric analysis performed to quantify the relative intensity of the indicated bands detected by Western blotting. (B) NS4A suppressed the ISRE reporter activity in IFNα or IFNβ stimulation. HEK293 T (5 × 10^4^) was transfected with Flag-NS4A (200 ng), luciferase reporter plasmid for ISRE (100 ng) and pRL-TK (5 ng), then stimulated with INF-α or IFN-β (50 ng/mL). The reporter activity was detected as Fig.1B. (C) Effect of NS4A on INF-α or IFN-β induced transcription of ISG15, ISG54 and ISG56 genes. HEK293 T (1 × 10^6^) was transfected with Flag-NS4A (2 μg) and then stimulated with INF-α or IFN-β (50 ng/mL). Levels of ISG15, ISG54, and ISG56 mRNAs were measured by real-time RT-PCR analysis, normalized to the cellular β-actin mRNA, and set in relation to mRNA levels of mock-infected cells. (D) Microscopy imaging of vector or NS4A transfected HEK293 T uninfected or infected with HSV-1-GFP (0.1 M.O.I.) or VSV-GFP (0.1 M.O.I.) for 24 h. Scale bar, 20 μm. (Right) Quantitative measurements of GFP in HEK293T cells infected with HSV-1-GFP or VSV-GFP. Student’s *t*-test was used for estimation of statistical significance. *, *P* < 0.05; **, *P* < 0.01 and ***, *P* < 0.001. Data are from three independent experiments. Mean values and standard deviations from three independent experiments are shown.

To further investigate whether TBEV interferes with molecules in the type I IFN signalling pathway, we analysed JAK/STAT activation in infected T98G cells. Immunoblot analysis indicated that steady-state levels of STAT1 and STAT2 were not affected by infection, while phosphorylation of STAT1 at Tyr701 and STAT2 at Tyr690 (p-STAT1 or p-STAT2) was greatly reduced in TBEV infection compared with VSV-infected cells (Figure 3(A)). Therefore, TBEV inhibited the STAT signalling pathway in response to IFN-β by preventing the phosphorylation of both STAT1 and STAT2.

To determine the ability of individual viral proteins to antagonize STAT activation, we generated plasmids encoding the individual TBEV structural or non-structural proteins C-terminally fused to Flag epitope tags and expressed them in T98G cells. The ISRE reporter analysis indicated NS4A and NS5 significantly inhibited STAT signalling (Supplementary Fig. 2A-B). Previous studies have also shown that NS5 blocks phosphorylation of STAT1 via hScrib [18]. We then focused on the effect of NS4A on type I IFN signalling. Overexpression of NS4A had no effect on the IFN-β production induced by SeV in HEK293 T cells (Supplementary Fig. 2C). ISRE reporter analysis indicated NS4A reduced promoter activity of ISRE after stimulation with IFN-α and IFN-β (Figure 3(B)). NS4A also impaired the transcription of ISGs, such as ISG15, ISG54 and ISG56, induced by IFN-α or IFN-β (Figure 3(C)). Overexpression of NS4A enhanced HSV and VSV replication in HEK293 T compared with the vector (Figure 3(D,E)). Taken together, these results suggest that TBEV-induced NS4A is an antagonist of IFN-mediated STAT signalling.

### NS4A inhibits the phosphorylation and dimerization of STAT1 / STAT2.

**Figure 4.**
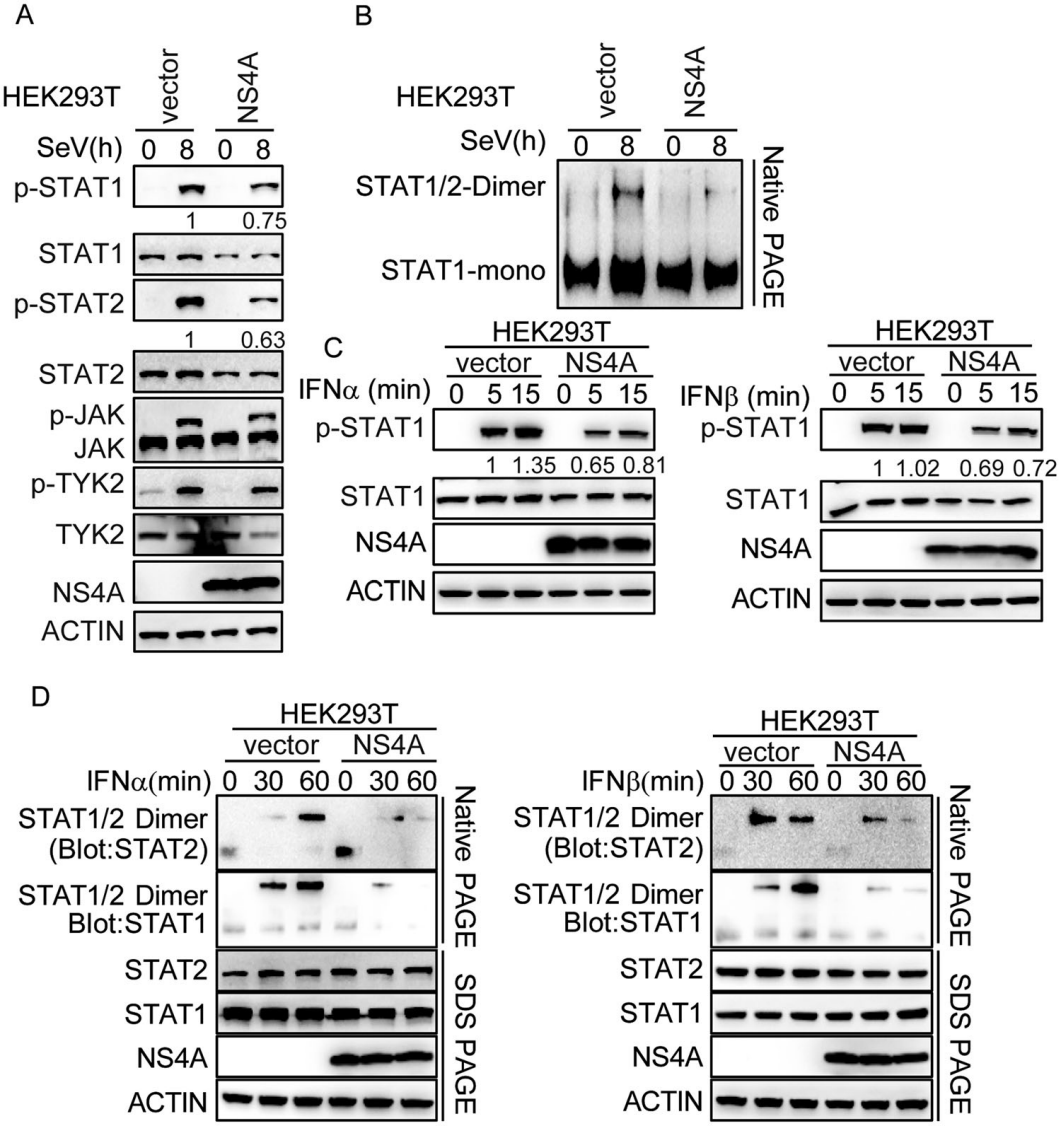
NS4A inhibits the phosphorylation and dimerization of STAT1/STAT2. (A&B) HEK293 T at 1 × 10^6^ mL was transfected with NS4A for 24 h then infected with mock or SeV for 8 h. (A)The total cell lysates were prepared and the tyrosine-phosphorylated STAT1 or STAT2 and phosphorylated JAK or TYK2 were determined by Western blotting. Meanwhile, the total amounts of STAT1, STAT2, JAK, TYK and NS4A were determined. ACTIN was used as a loading control. (B) Native PAGE analysis of STAT1/2 in dimer or monomer form. (C&D) HEK293 T at 1 × 10^6^ mL was transfected with NS4A for 24 h then treated with INF-α or IFN-β (50 ng/mL). The total cell lysates were prepared at indicated time. (C) The phosphorylated STAT1 or STAT2 and total STAT1, STAT2 and NS4A were determined by Western blotting. ACTIN was used as a loading control. The numbers in A&C means the relative intensity of the p-STAT1/ STAT1 or p-STAT2/STAT2 detected by Western blotting. (D) The dimer of STAT1/2 was analysed by Native PAGE. Data are representative of three independent experiments.

We then explored the mechanism through which NS4A suppresses STAT signalling. First, we found NS4A had no effect on the expression of IFNR1 or IFNR2 (Supplementary Fig. 3). Next, we detected whether NS4A could prevent the phosphorylation of both STAT1 and STAT2 in the context of TBEV infection. HEK293 T cells were transfected with NS4A and then challenged with SeV. Western blot analyses showed that p-STAT1 and p-STAT2 were significantly decreased and the dimerization of STAT1/STAT2 was barely detectable in cells expressing the NS4A fusion protein in HEK293 T or THP-1 (Figure 4(A,B), Supplementary Fig. 4A and B). Examination of STAT activation by Western blotting or native PAGE following stimulation with IFN-α or IFN-β revealed that both phosphorylation and dimerization were inhibited in NS4A-overexpressing HEK293 T cells (Figure 4(C,D)). This suggests that NS4A suppresses phosphorylation and dimerization of STAT1/STAT2.

### NS4A suppresses the formation of ISGF3

**Figure 5.**
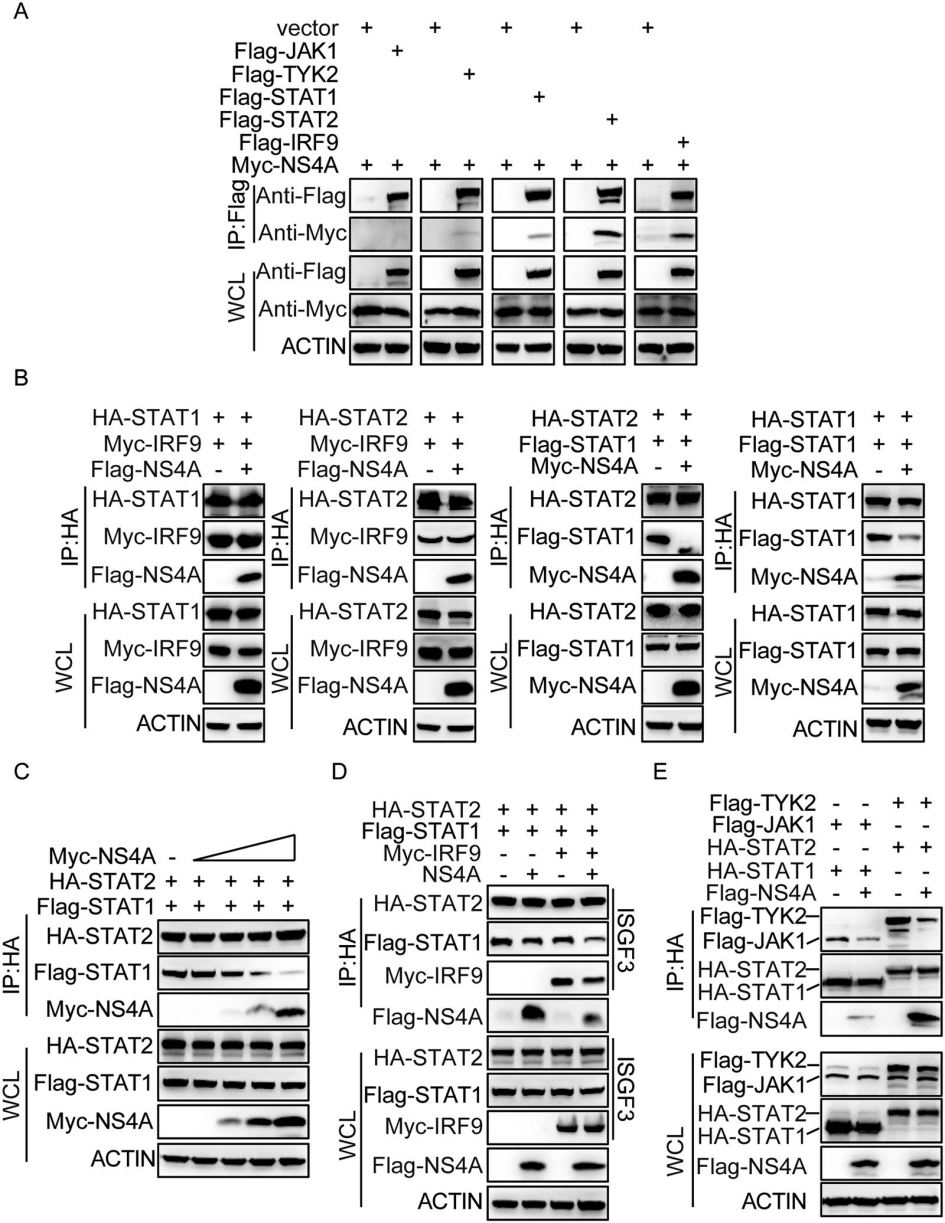
NS4A suppresses the formation of ISGF3. (A) The interaction of NS4A with JAK1, TYK2, STAT1, STAT2 or IRF9. (A) The interaction of NS4A with JAK1, TYK2, STAT1, STAT2 or IRF9. IP and IB analyses of lysates of HEK293 T cells (1 × 10^6^) overexpressing plasmids encoding Myc-tagged NS4A or Flag-JAK1, Flag-TYK2, Flag-STAT1, Flag-STAT2 or Flag-IRF9. (B) The effect of NS4A present on the interaction between the pairings of ISGF3 complex. IP and IB analyses the association of STAT1, STAT2 and IRF9 in the presence or absence of Flag-NS4A. (C) IP and IB analyses the interaction of STAT1 and STAT2 when Flag-NS4A gradient overexpression. (D) IP and IB analyses the formation of ISGF3 in the presence or absence of Flag-NS4A. (E) IP and IB analyses the interaction of TYK2 with STAT1 or the interaction of JAK1 with STAT2 in the presence or absence of Flag-NS4A. Data are representative of three independent experiments.

It has been reported that tyrosine-phosphorylated STAT1 and STAT2 heterodimerize and join with IRF9 to form the transcription factor complex ISGF3 [26]. To elucidate the mechanism of NS4A-mediated inhibition STAT activation, we investigated the interaction of NS4A with a component of ISGF3. Immunoprecipitation results showed that NS4A interacted with STAT1, STAT2 and IRF9 (Figure 5(A)). NS4A had no effect on the interaction of STAT1 with IRF9 and STAT2 with IRF9, but impaired the interaction of STAT1 with itself or STAT2 (Figure 5(B)) in a dose dependent manner (Figure 5(C)). In addition, NS4A impaired the formation of ISGF3 (Figure 5(D)). We then analysed whether NS4A could affect the interaction of STAT1 or STAT2 with the downstream kinases, JAK1 or TYK2. Immunoprecipitation analysis indicated that NS4A impaired the interaction of STAT1 and STAT2 with their downstream kinases (Figure 5(E)). Taken together, these results indicate that NS4A interferes with the interaction of STAT1 and STAT2 to disrupt the formation of ISGF3.

### NS4A interacts with the SH2 and TAD domain of STAT1 or STAT2 to impair their phosphorylation

**Figure 6.**
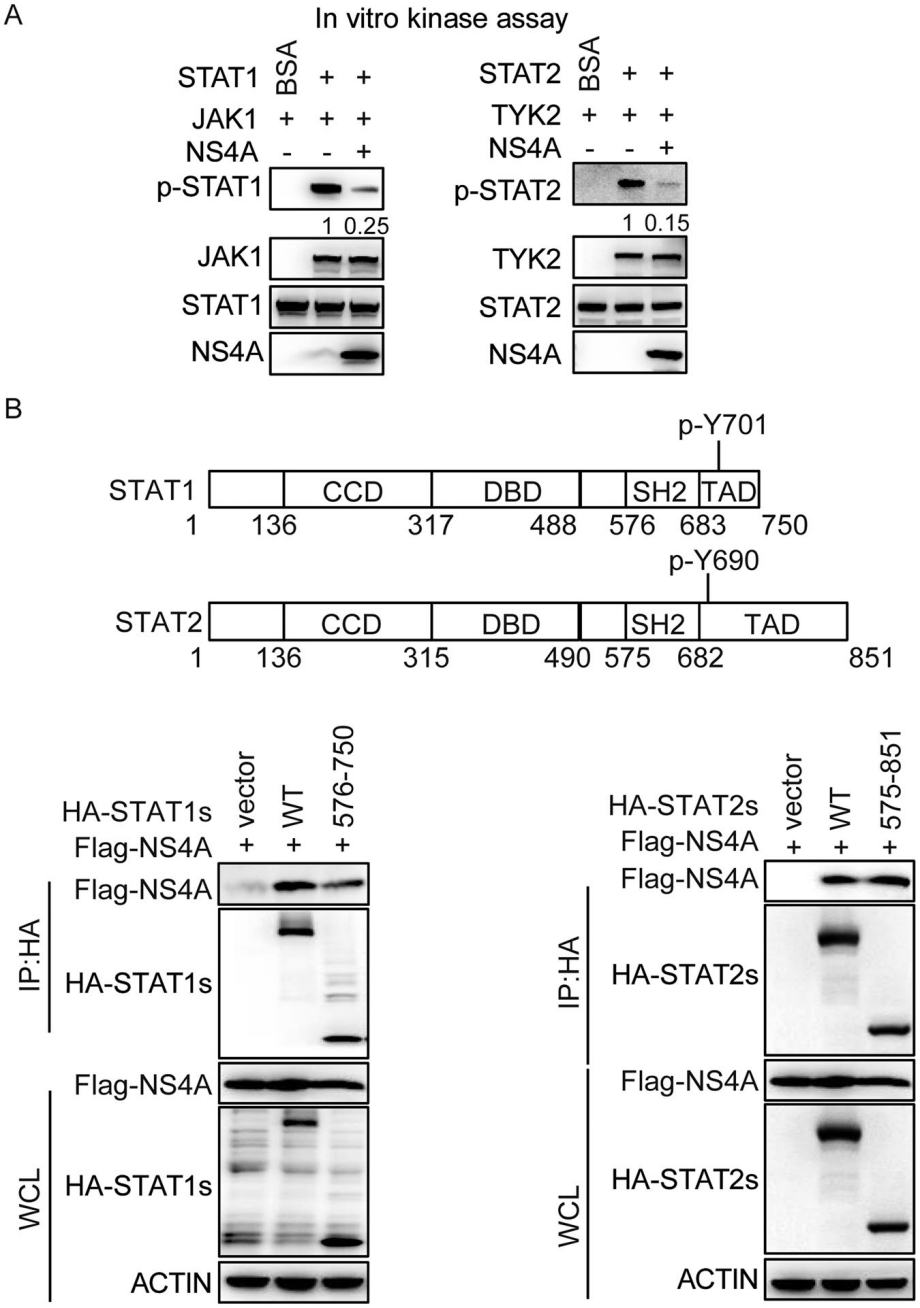
NS4A interacts with the SH2 and TAD domain of STAT1 or STAT2 to impair their phosphorylation. (A) *In vitro* kinase assay of STAT1 phosphorylated at Tyr701 (Y701) and STAT2 phosphorylated at Tyr690 (Y690). The peptide of STAT1 or STAT2 and their kinase JAK1 or TYK2 were introduced into a mixture containing Flag-NS4A. (B) Schematic representation of STAT1 or STAT2 (top). IB analysis of HEK293 T cells co-transfected for 48 h with Flag-NS4A and HA-wild-type STAT1 or STAT2, or their mutants, assessed with whole-cell lysates (10% input) or IP with anti-HA antibody (bottom). The numbers in A means the relative intensity of the p-STAT1/ STAT1 or p-STAT2/STAT2 detected by Western blotting. Data are representative of three independent experiments.

Since NS4A inhibited the phosphorylation of STAT1/STAT2, we hypothesized that NS4A blocked the interaction of the STAT1/STAT2 with downstream kinases by competitive binding reactions with STAT1/STAT2. To test this hypothesis, we expressed NS4A, STAT1, STAT2 and the downstream kinases JAK1 and TYK2 and performed an *in vitro* kinase assay. The results indicated that NS4A significantly decreased phosphorylation of STAT1 or STAT2 by JAK1 or TYK2 (Figure 6(A)). Given that the phosphorylation sites of STAT1 or STAT2 are located in the SH2 and TAD domains, we speculated that NS4A might interact with this domain. The co-immunoprecipitation assay showed that the SH2 and TAD domains of STAT1 or STAT2 indeed interacted with NS4A (Figure 6(B)).

### The ubiquitination of NS4A at Lys132 is important for the interaction of NS4A and STAT1

**Figure 7.**
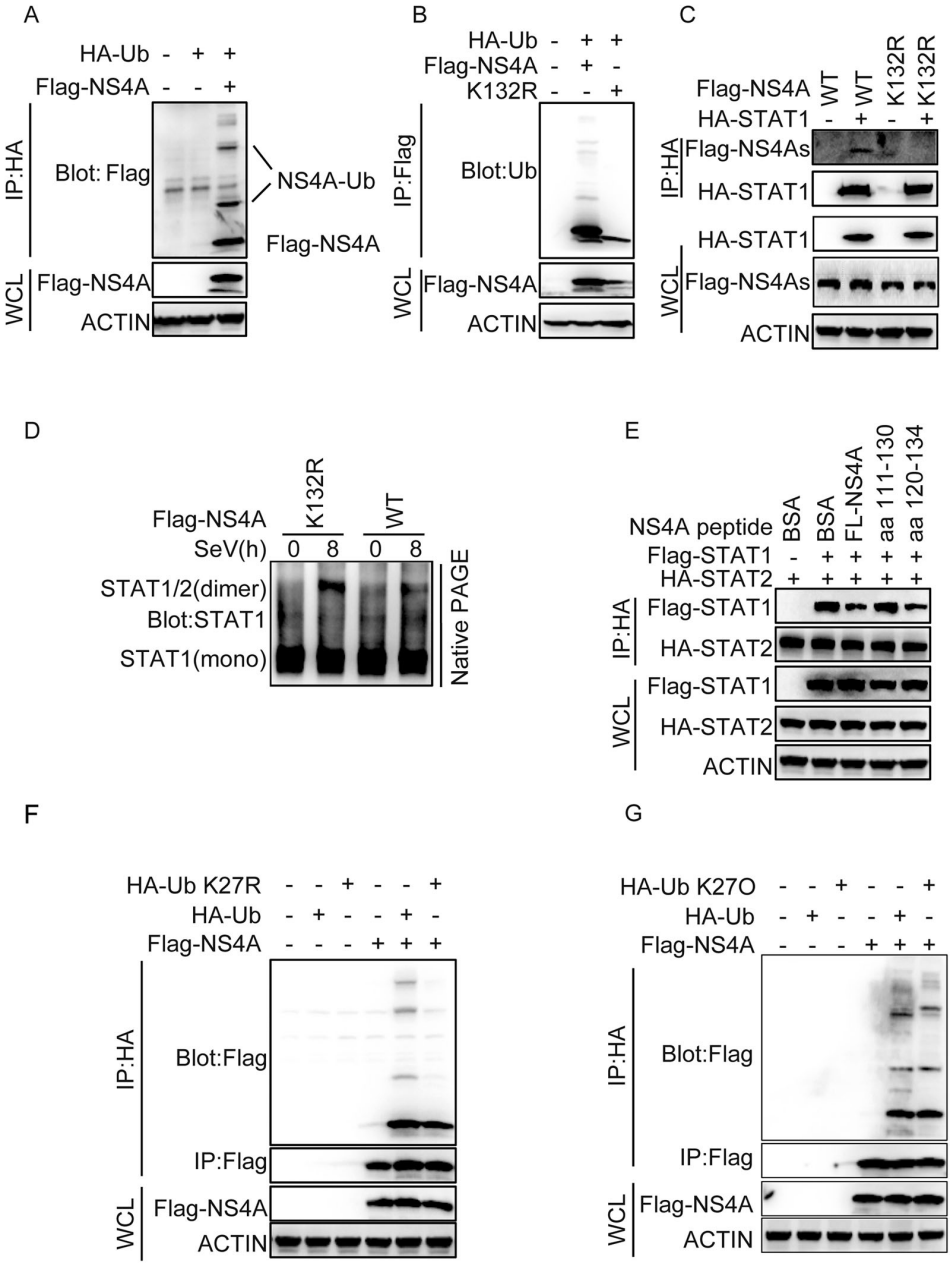
The ubiquitination of NS4A at Lys132 is important for the interaction of NS4A and STAT1. (A) HEK293 T cells (1 × 10^6^) were transfected with the indicated plasmids. 24 h after transfection, ubiquitination assays and immunoblotting analysis were performed with the indicated antibodies. (B) Effect of K132R mutant on ubiquitination of NS4A. The experiments were performed as in A except that the indicated plasmids were used. (C) Effect of K132R mutant on the interaction of NS4A with STAT1. HEK293 T cells (1 × 10^6^) transfected with the indicated plasmids (1 μg each) for 36 h followed by co-immunoprecipitation experiments and immunoblotting analysis with the indicated antibodies. (D) Effect of K132R mutant on the dimer of STAT1 and STAT2. The cells were performed as in C. The dimer of STAT1/2 was analysed by Native PAGE. (E) Effect of NS4A peptide on the interaction of STAT1 and STAT2. HEK293 T cells (1 × 10^6^) were transfected with Flag-STAT1 and HA-STAT2. 48 h after transfection, the cells were lysed and then added the different peptides of NS4A, IP analyses the interaction of STAT1 with STAT2. (F&G) Immunoblot analysis of the ubiquitination of NS4A in T98G cells co-transfected with HA-Ub, HA-Ub-K27R (F) or HA-Ub-K27O (G). Data are representative of three independent experiments.

We then sought to understand how NS4A interfered with the interaction of STAT1 and STAT2. Bioinformatics analysis highlighted that the K132 could be ubiquitinated or SUMOylated. Comparing the sequences of fragments from different subtypes of TBEV, LGTV (Langat virus) and LIV (Louping ill virus), showed high conservation as evident by the lysine residue at position 132 (Supplementary Fig. 5A). We found that NS4A could be ubiquitinated (Figure 7(A)). The lysine residues were mutated into arginine (K to R) to mimic de-ubiquitination (K132R) followed by transient transfection and immunoprecipitation assays. The K132R mutation abolished the ubiquitination of NS4A (Figure 7(B)) and diminished the interaction of STAT1 and NS4A (Figure 7(C)). In a native PAGE assay, compared with the WT, the K132R mutation had little effect on the dimerization of STAT1 and STAT2 (Figure 7(D)). To further confirm K132 was important for the interaction of STAT1 and NS4A, we expressed full-length NS4A and peptides consisting of 15-amino acid (aa120–134) or 20-amino acid (aa111–130) *in vitro* and performed immunoprecipitation assays. The peptide (aa120–134) harboring K132 impaired the interaction of STAT1 and STAT2 similar to the full-length sequence (Figure 7(E)). These data suggest that K132 residue in NS4A was important for the blocking the dimerization of STAT1 and STAT2. Next, we determined the mechanism by which ubiquitin was linked to NS4A in T98G cells via the established site of isopeptide linkage (K27R). When K27R ubiquitin was added, the abundance of the slowest migrating bands decreased. This effect was due to capping of poly-ubiqutin chains by the mutant ubiquitin. Moreover, we compared the mechanism underlying ubiquitin linkage of NS4A and found that polyubiquitin linkage of NS4A was through HA-ubquitin wild-type and mutant in all Lys except Lys 27 (K27O) (Figure 7(F,G)). Together, these data indicate the polyubiquitin chains attached to NS4A were linked through Lys 27. Lys 27-linked ubiquitin chains were the preferred point of contact or binding of NS4A to STAT1.

### The physiological significance of ubiquitination of NS4A

**Figure 8.**
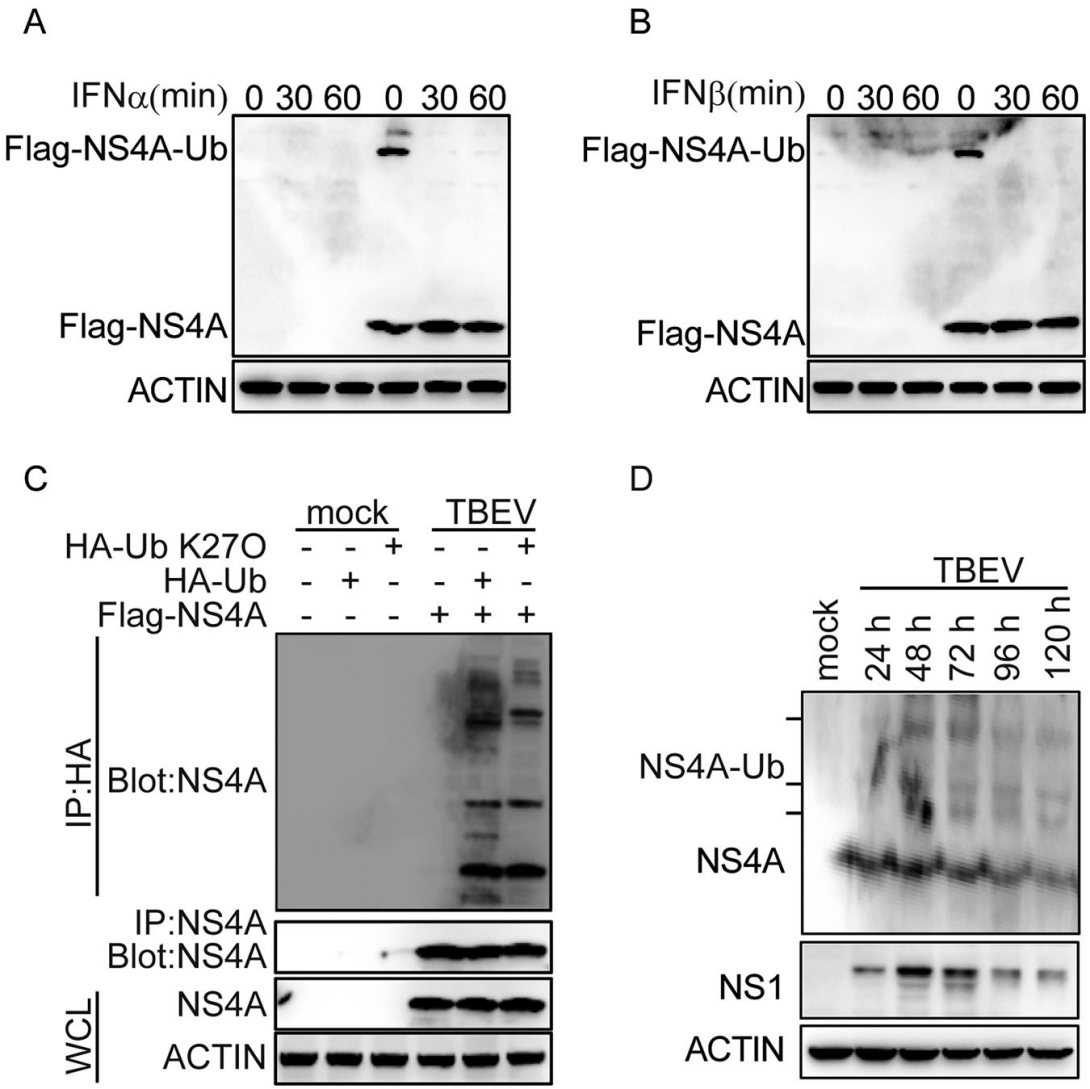
The physiological significance of ubiquitination of NS4A. (A&B) Immunoblot analysis of the ubiquitination of NS4A in T98G cells transfected with Flag-NS4A and then stimulated with human INF-α (A) or IFN-β (B) (50 ng/mL) for the indicated times. (C) Immunoblot analysis of the ubiquitination of NS4A in T98G cells co-transfected with HA-Ub or HA-Ub-K27O and then infected or uninfected by TBEV for 48 h. (D) Immunoblot analysis of the ubiquitination of NS4A in T98G cells with TBEV infection for the indicated times. Data are representative of three independent experiments.

Ubiquitination of NS4A was important for preventing the activation of STAT signalling. To investigate the regulation of NS4A ubiquitination, we detected the modification of NS4A in IFN treatment or TBEV infection. In IFN-α or IFN-β treatment, the ubiquitination of NS4A was largely abolished in T98G cells (Figure 8(A,B)). But during TBEV infection, ubiquitination, particularly the Lys27, was present in NS4A (Figure 8(C)). The ubiquitination of NS4A was enhanced at 48 and 72 h and then weakened at 96 and 120 h after TBEV infection (Figure 8(D)). This was consistent with the trend in the expression of NS1 or NS4A. These results suggest that TBEV infection could enhance the ubiquitination of NS4A, but IFN suppressed this modification.

## Discussion

In this paper, we have identified TBEV NS4A as an IFN antagonist that associates with STAT1/STAT2, a necessary component of the ISGF3 transcription complex. While the expression of TBEV NS4A alone suppressed STAT1/STAT2 binding, the ability of TBEV NS4A to target STAT1/STAT2 for phosphorylation and dimerization required NS4A ubiquitination. TBEV infection promoted Lys27-linked ubiquitination of NS4A. Our findings revealed that TBEV NS4A was also a potent IFN antagonist and may reflect a very straightforward process of TBEV to the host IFN systems.

Neuroinvasiveness and neurovirulence are key steps in the pathogenesis of neurotropic viruses in TBEV infection. Protection against infection requires coordinated action of the type I IFN system in both peripheral and central nervous system to prevent TBEV-induced inflammation and development of encephalitis [27]. It has been reported that IPS1, a key adaptor molecule that acts downstream of RLRs (RIG-I and MDA5), activates the type I IFN and NF-κB pathways in TBEV infection [24]. In our studies, we showed RIG-I and MDA5 but not TLR3 recognized TBEV RNA to induce IFN-β and ISRE activation in T98G cells. In agreement with our studies, TBEV activated IRF-3 signalling pathway dependent on RIG-I/MDA5 [28]. Previous studies have shown that TBEV induces IFN-β mRNA through RIG-I, similarly to JEV, and not through MDA5 in human osteosarcoma (U2OS) cells [23]. Collectively these results confirm that, RIG-I-like receptors play an important role in recognizing TBEV RNA and activation of IFN-β.

The type I IFN system is an important component of innate immunity and limits the viral load of many flavivirus infections. A number of studies have reported that flaviviruses’ non-structural proteins have evolved distinct strategies to counteract an IFN response. While non-structural proteins other than NS5 for WNV, DENV and KUNV have been implicated in JAK-STAT interference, NS5 is the main virulence factor for JEV and LGTV [15,18,28–30]. In TBEV infection, slow accumulation of IFN-β mRNAs and delay of the transcriptional ISGs suggest TBEV negatively regulates the type I IFN pathway. The fact that expression of TNF−α and IL-6 (NF−κB driven genes) were not affected, suggested that inhibition was not due to virus-mediated cell cytotoxicity or a general suppression of receptor-mediated signal transduction. It has been reported TBEV NS5 protein has a functional implication for IFN signalling via hScrib [18]. Here, we found NS4A could block the phosphorylation and dimerization of STAT1/STAT2. This is the first time it has been reported that NS4A displayed antagonism for IFN-STAT signalling.

We found that ubiquitination of TBEV NS4A was critical for the inhibition for STAT signalling. NS4A could be modified by ubiquitination, and the K132R mutation abolished the ubiquitination of NS4A and the interaction of STAT1 and NS4A. In addition, the K132R mutation could reduce the expression of NS4A. These results suggest that the ubiquitination of NS4A was essential for suppressing STAT activation.

During TBEV infection, NS4A could be modified by ubiquitin. However, following treatment with IFN-α or IFN-β, ubiquitination of NS4A was largely abolished and NS4A no longer inhibited activation of STAT. It could be explained that the IFN-β pre-treatment resulted in reduced production of TBEV [31]. During TBEV infection, ubiquitination of NS4A was strengthened and then weakened with the accumulation of type I IFN and the stimulation of ISGs. This showed the antagonism of natural immunity against the TBEV infection. We found that the K132R mutation of TBEV NS4A weakened interaction with STAT1, whereas the K132R mutation promoted ubiquitination, particularly the Lys27-linked ubiquitination. These results imply that Lys27-linked ubiquitination of NS4A was an indispensable part of the innate immune escape of TBEV.

In conclusion, we have identified that RIG-I/MDA5 recognized TBEV RNA but not TLR3. We further reveal that NS4A is critical for suppressing the phosphorylation and dimerization of STAT1/STAT2 as an IFN-STAT antagonist. Lys27-linked ubiquitination at K132 of NS4A is necessary for the inhibition of STAT activation. TBEV infection could enhance the ubiquitination of NS4A at K132, but type I IFN treatment could suppress this modification. Our findings highlight the significance of TBEV NS4A against host innate immunity, providing a molecular explanation for the innate immune escape of TBEV and a potential intervention target.

## Supplementary Material

Supplemental Material
