# Supplementary material for: Tick-borne encephalitis virus NS4A ubiquitination antagonizes type I interferon-stimulated STAT1/2 signalling pathway
Source: Emerg Microbes Infect. 2020 Mar 27;9(1):714–26. doi: 10.1080/22221751.2020.1745094 (PMC7170394; doi:10.1080/22221751.2020.1745094)
Supplement: Supplemental Material [file TEMI_A_1745094_SM8408.docx]

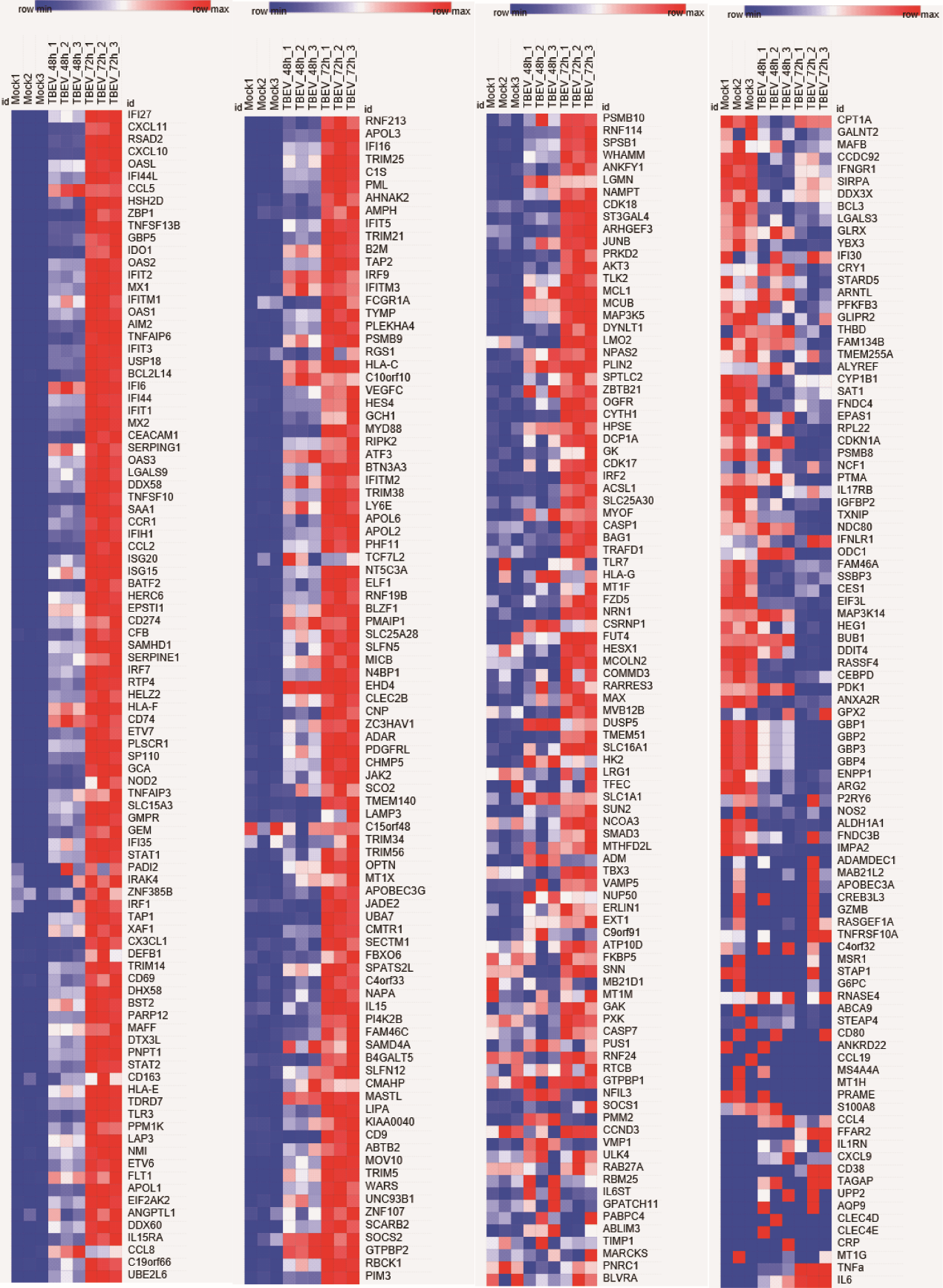


**Supplementary Figure 1. Overview of differentially expressed interferon simulated genes (ISGs)** **in TBEV infection.** T98G cells were infected with TBEV MOI=1) at indicated times. Three independent biological replicates were included for each of the combinations [untreated mock cells (control); cells infected with TBEV for 48 h; cells infected with TBEV for 72 h]. Total cellular RNA was isolated and used for transcriptome analysis. List of ISGs in TBEV infection. The minimum value of the rows was in blue and the max was in red.


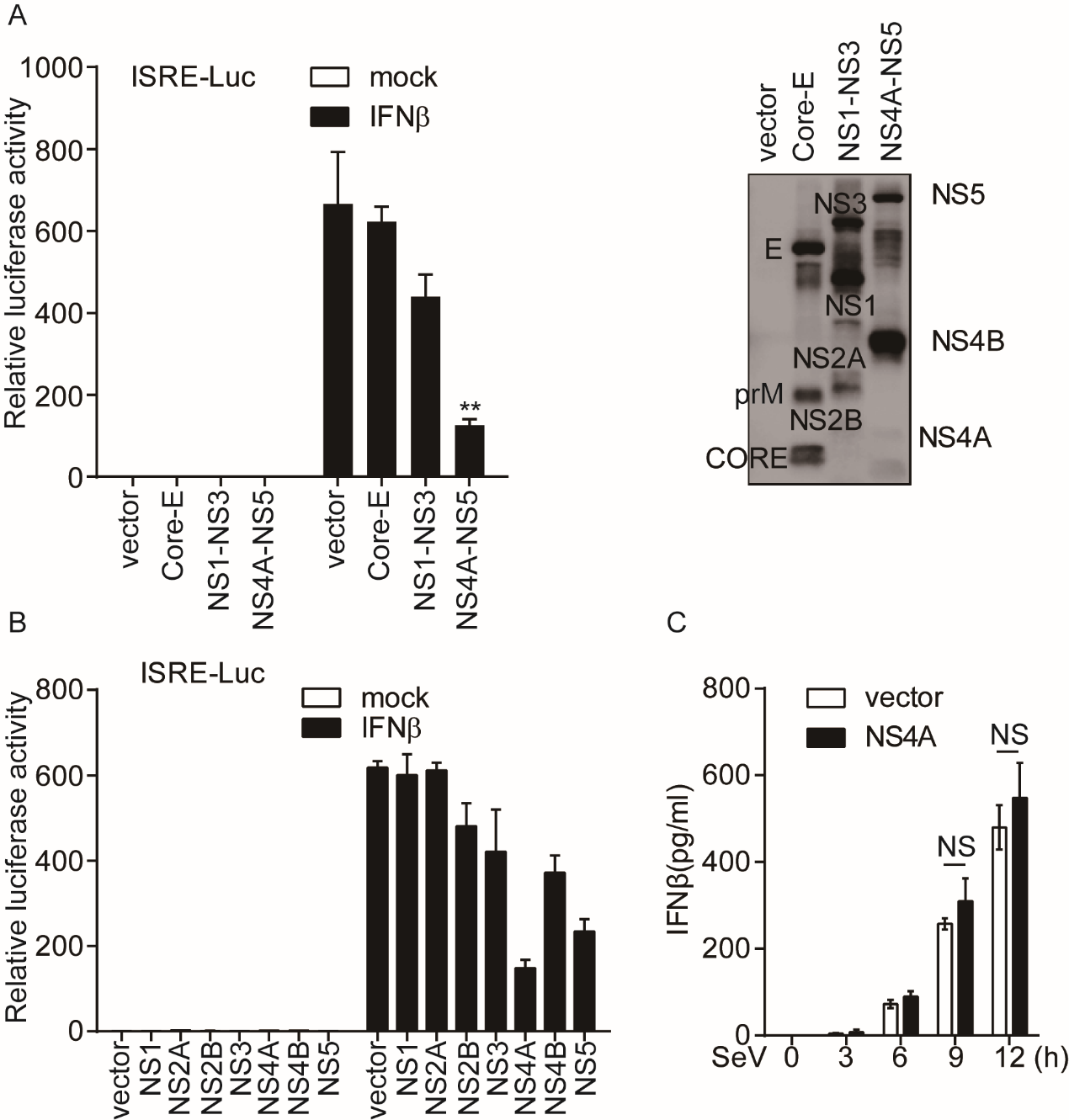


**Supplementary Figure 2.** **TBEV NS4A is responsible for inhibiting the activity of ISRE.** (A) Identification of TBEV proteins that inhibit ISRE activity. T98G cells in 48-well plates were co-transfected with 200 ng Flag-tagged plasmids encoding TBEV proteins or empty vector together with 100 ng pGL2-ISRE-Luc and 10 ng phRL-TK. Stimulated with IFN-β for 24 h, cells were harvested to measure luciferase activity. The expression of TBEV proteins was analyzed by Western blotting using an anti-Flag Ab. An asterisk indicates TBEV proteins. (B) Identification of TBEV non-structure proteins that inhibit ISRE activity. T98G cells in 48-well plates were co-transfected with 200 ng Flag-tagged plasmids encoding TBEV non-structure proteins or empty vector together with 100 ng pGL2-ISRE-Luc and 10 ng phRL-TK. Stimulated with IFN-β for 24 h, cells were harvested to measure luciferase activity. (C) The effect of NS4A for IFN-β production. HEK293T in 48-well plates were transfected with 200 ng Flag-tagged-NS4A and the infected by SeV. The culture supernatants were harvested at indicated times and the IFN-β was detected by ELISA. Student's t-test was used for estimation of statistical significance. NS, there was no significant difference and **, P < 0.01. Data are from three independent experiments. Mean values and standard deviations from three independent experiments per group.


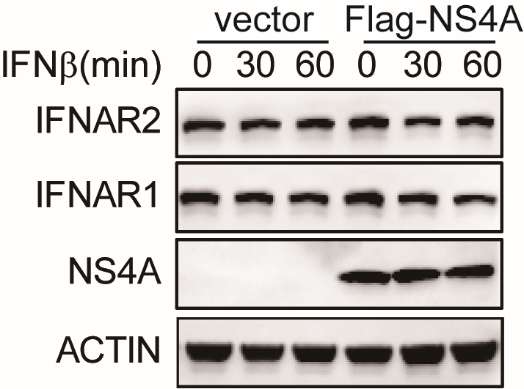


**Supplementary Figure 3.** T98G cells (1×10^6^) were transfected with Flag-tagged-NS4A (2 μg) for 24 h then treated with IFN-β (50 ng/mL). Immunoblot analyses the expression of IFNR1 and IFNR2 in the presence or absence of Flag-NS4A during IFN-β challenge. Data are representative of three independent experiments.


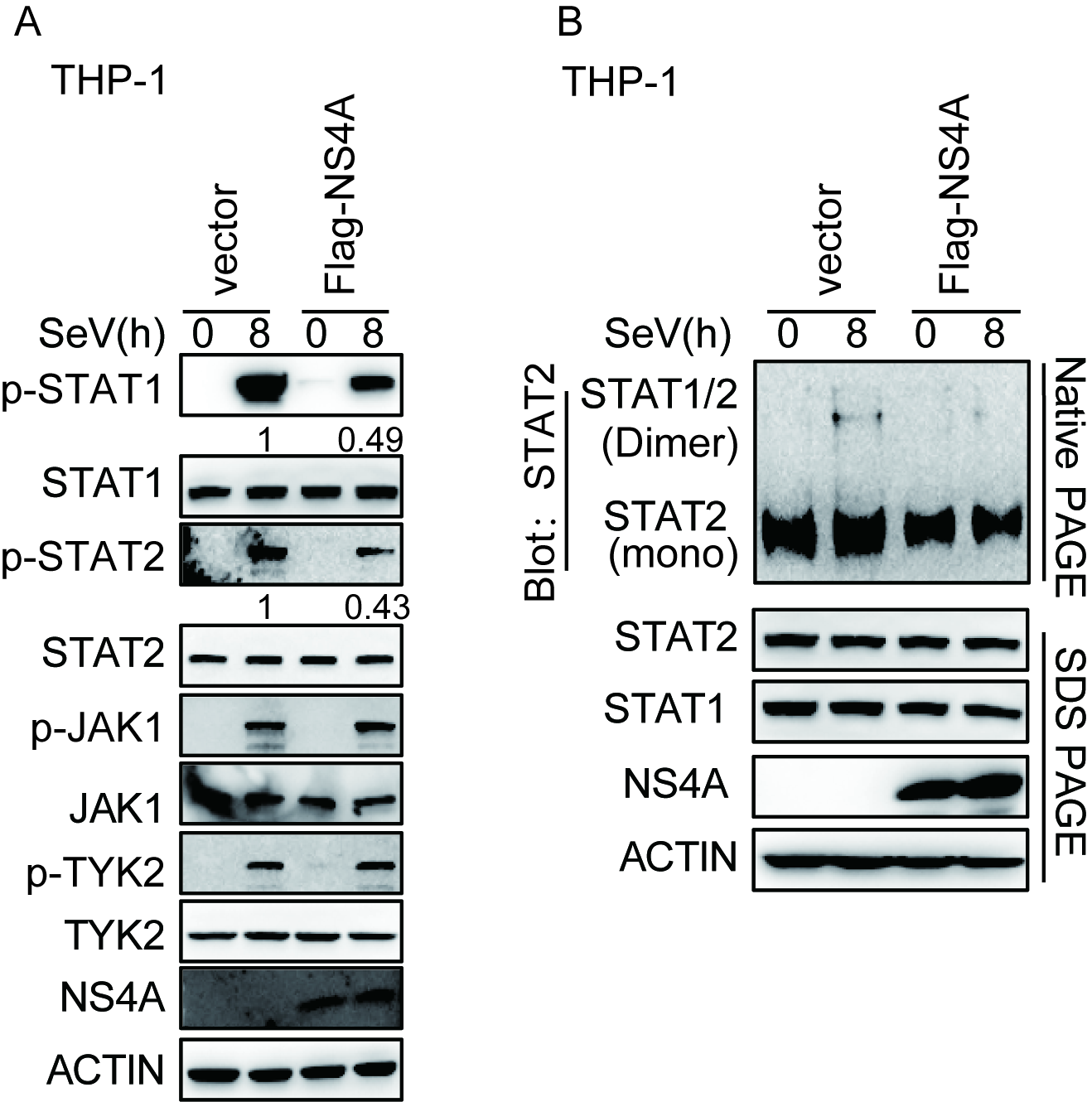


**Supplementary Figure 4. NS4A inhibits the phosphorylation and dimerization of STAT1 / STAT2.** (A&B) The human acute monocytic leukemia cell line (THP-1, 1×10^6^) was expressed with NS4A by Lentivirus expression system for 24 h then infected with mock or SeV for 8 h. (A)The total cell lysates were prepared and the tyrosine-phosphorylated STAT1 or STAT2 and phosphorylated JAK or TYK2 were determined by Western blotting. Meanwhile, the total amounts of STAT1, STAT2, JAK, TYK and NS4A were determined. ACTIN was used as a loading control. The numbers in A means the relative intensity of the p-STAT1/ STAT1 or p-STAT2/STAT2 detected by Western blotting. (B) Native PAGE analysis of STAT1/2 in dimer or monomer form. Data are representative of three independent experiments.


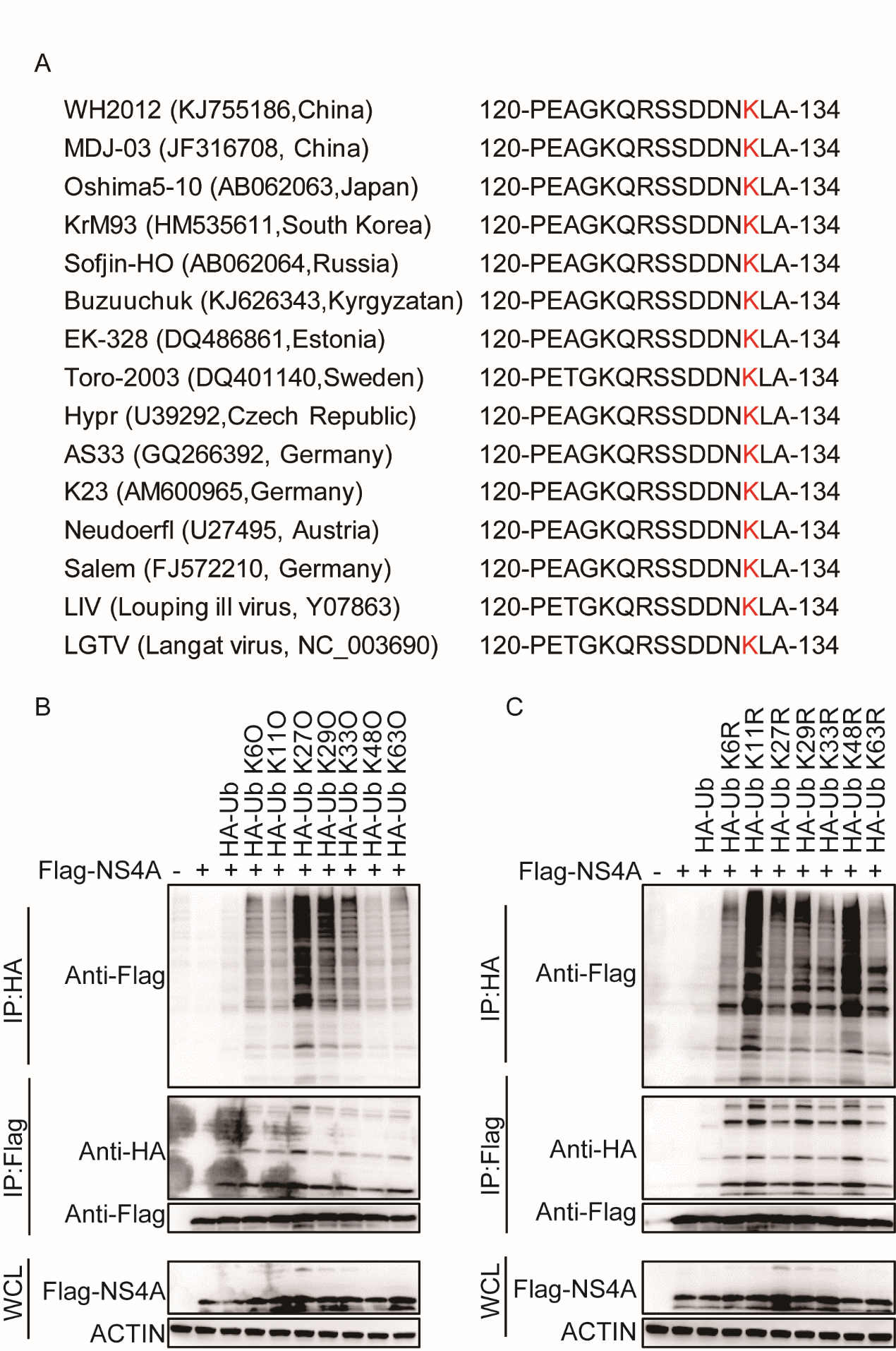


**Supplementary Figure 5.** (A) Sequence alignment using clustalx of aa120-134 of NS4A with the sequence reported as various strains of TBEV, Louping ill virus (LIV) or Langat virus (LGTV). (B&C) Ub-linkage-specific effects on the ubiquitination of NS4A. T98G cells were transfected with Ub variants with mutant in all Lys except the indicated lysine (K6O, K11O, K27O, K29O, K33O, K48O or K63O) or specific lysine mutated to arginine (K6R, K11R, K27R, K29R, K33R, K48R or K63R) for 24 h. Immunoblot analysis of the ubiquitination of NS4A. Data are representative of three independent experiments.

**Supplementary Table 1. Primers for genes amplification**

| Gene | Orientation | | | Sequence (5’-3’) |
| --- | --- | --- | --- | --- |
| NS4A | | F | GTTTAAACATGAGCTTTGGAGATGTGTTGAGCGG | |
| NS4A | | R | ACTAGTTTACAGATCCTCTTCAGAGATGAGTTTCTGCTCGGCGGCTACCAGTCCAGCTAGACTGC | |
| NS4A (K132R) | | F | AAGCTTAGCTTTGGAGATGTGTTGAGCGG | |
| NS4A (K132R) | | R | GGATCCTTAGGCGGCTACCAGTCCAGCTAGACTGCAGAGCGTCAACAGGAAGTAGGCCAACCTGTTG | |
| JAK1 | | F | GCGGCCGCCATGCAGTATCTAAATATAAAAGAGG | |
| JAK1 | | R | GGTACCTTATTTTAAAAGTGCTTCAAATCC | |
| TYK2 | | F | GCGGCCGCCATGCCTCTGCGCCACTGGGGGATG | |
| TYK2 | | R | GGTACCTCAGCACACGCTGAACACTGAAGG | |
| IRF9 | | F | GCGGCCGCATGGCATCAGGCAGGGCACGCTG | |
| IRF9 | | R | GGTACCCTACACCAGGGACAGAATGGCTG | |
| STAT1 | | F | GCGGCCGCCATGTCTCAGTGGTACGAACTTCAGC | |
| STAT1 | | R | GGTACCCTATACTGTGTTCATCATACTGTCG | |
| STAT1/Δ1-575 | | F | GCGGCCGCCATGGGGTGCATCATGGGCTTCATCAGC | |
| STAT2 | | F | GCGGCCGCCATGGCGCAGTGGGAAATGCTG | |
| STAT2 | | R | GGTACCCTAGAAGTCAGAAGGCATCAAGGG | |
| STAT2/Δ1-574 | | F | GCGGCCGCCATGGGACGCATCATGGGCTTTGTGAGTC | |

F indicate forward primers; R indicate reverse primers; Red color denote Pme I (forward), Hind III (forward), Not I (forward), Spe I (reverse), BamH I (reverse) or Kpn I (reverse) restriction site. Blue color denotes termination codon.

**Supplementary Table 2. Primers for mRNA Quantification**

| Gene name | Orientation | | | Sequence (5’-3’) |
| --- | --- | --- | --- | --- |
| IFNB1 | | F/R | AGGACAGGATGAACTTTGAC/TGATAGACATTAGCCAGGAG | |
| ISG15 | | F/R | GAGAGGCAGCGAACTCATCTT/CCAGCATCTTCACCGTCAGG | |
| ISG54 | | F/R | GGTCTCTTCAGCATTTATTGGTG/TGCCGTAGGCTGCTCTCCA | |
| ISG56 | | F/R | TAGCCAACATGTCCTCACAGAC/TCTTCTACCACTGGTTTCATGC | |

F indicate forward primers; R indicate reverse primers.
